# Stress and Cardiometabolic Disease Risk for Indigenous Populations throughout the Lifespan

**DOI:** 10.3390/ijerph18041821

**Published:** 2021-02-13

**Authors:** Melissa E. Lewis, Hannah I. Volpert-Esmond, Jason F. Deen, Elizabeth Modde, Donald Warne

**Affiliations:** 1 Department of Family and Community Medicine, School of Medicine, University of Missouri, Columbia, MO 65212, USA; Eam7c9@health.missouri.edu; 2 Psychology Department, University of Texas, El Paso, TX 79902, USA; Hivolpertes@utep.edu; 3 Department of Pediatrics, University of Washington, Seattle, WA 98195, USA; jason.Deen@Seattleschildrens.org; 4 Family & Community Medicine Department, University of North Dakota, Grand Forks, ND 58202, USA; Donald.Warne@und.edu

**Keywords:** Indigenous population, stress, cardiometabolic disease, historical trauma, adverse childhood experiences

## Abstract

Background: Indigenous people experience the greatest cardiometabolic disease disparity in the Unites States, yet high cardiometabolic disease risk factors do not fully explain the extent of the cardiometabolic disease disparity for Indigenous people. Stress, trauma, and racism occur at high rates within Indigenous communities and have not been well explored as significant contributors to cardiometabolic disease disparities despite emerging literature, and therefore will be described here. Methods: This descriptive study explores the relationship between cardiometabolic disease risks and Indigenous-specific stressors (e.g., early childhood stress and trauma, adulthood stress and trauma, and historical and intergenerational trauma) using current literature. Indigenous-specific protective factors against cardiometabolic disease are also reviewed. Results. Increasing research indicates that there is a relationship between Indigenous-specific stressful and traumatic life experiences and increased cardiometabolic disease risk. Mental health and psychophysiology play an important role in this relationship. Effective interventions to reduce cardiometabolic disease risk in Indigenous communities focus on ameliorating the negative effects of these stressors through the use of culturally specific health behaviors and activities. Conclusions: There is increasing evidence that cultural connection and enculturation are protective factors for cardiometabolic disease, and may be galvanized through Indigenous-led training, research, and policy change.

## 1. Introduction

In the United States, cardiovascular disease (CVD) is the leading cause of death for Indigenous peoples. (We capitalize and use the word Indigenous to refer to Indigenous communities around the world. When a certain population is referenced, the specific group or tribe name will be used (e.g., American Indian, First Nations, and Cherokee Nation). We will also use the group name that each study uses for specificity, resulting in several different names used for different Indigenous groups.) Indigenous people have a higher prevalence of and greater morbidity and mortality from CVD compared to all other racial categories [1,2,3,4,5]. This is due, in part, to an increased prevalence of cardiometabolic disease (CMD) risk factors. Cardiometabolic syndrome or disease (CMD) refers to risk factors such as elevated blood sugar, abdominal obesity, hypertension, dyslipidemia, and elevated triglycerides, resulting in an interrelated set of conditions that can include obesity, and hypertension, type two diabetes mellitus (T2D), stroke, and even CVD. Compared to the general population, Indigenous people disproportionately experience hypertension, obesity, type two diabetes mellitus (T2D) or prediabetes, smoking, and sedentary lifestyle [6,7,8,9,10,11]. Especially alarming is the increased prevalence of CVD risk factors in Indigenous youth [12]. Indigenous adolescents have a high prevalence of CVD risk factor clusters, with 24.9% meeting formal criteria for metabolic syndrome, defined as obesity with associated hypertension, dyslipidemia and/or insulin resistance. This collection of risk factors is associated with excess mortality, a two-fold risk of atherosclerotic CVD, and a five-fold risk of developing T2D in adulthood [13,14,15,16,17,18].

Disproportionate exposure to historical and contemporary stress and psychological trauma among Indigenous people in the context of CVD is understudied, despite a robust evidence base showing a relationship between increased stress and incidence of CVD and CMD in the general population. The current paper briefly reviews existing research on the stress–cardiometabolic disease relationship among the general population, extends existing research by considering stressors experienced uniquely by Indigenous people and how these stressors contribute to CMD, and lastly, presents promising solutions and interventions to address CMD disparities experienced by Indigenous people. 

### Stress–CMD Relationship

Within the general population, stress is an important contributor to CMD risk. For example, Steptoe and Kivimäki have reported that chronic stress is associated with a 40–60% excess risk of development of coronary heart disease [19,20]. Both past stress (e.g., childhood trauma) and concurrent stress (e.g., daily hassles, work strain, marital strain, and social isolation) have been linked to T2D [21], arterial stiffness [22], and ischemic heart disease [23,24,25]. As proposed in the stress–health–illness model [26], stress leads to CMD risk factors and negative outcomes through a causal path that reflects autonomic nervous system dysfunction and which is mediated by the physiological effects of acute and chronic stress [27]. Specifically, chronic stress increases allostatic load and disrupts normal systems of arousal, including immune [28], neuroendocrine [29], and cardiovascular functioning [30,31,32,33]. Numerous systems are involved in this dysregulation, including the hypothalamic–pituitary–adrenal (HPA) axis, which performs a central function in organizing the neuroendocrine response to stress, and the sympathetic–adrenal–medullary (SAM) system, which works in concert with the autonomic nervous system to regulate cardiovascular, pulmonary, and immune systems [27,34,35,36]. Increased inflammation as a result of the dysregulation of these systems is associated with increased mortality and CVD risk [36,37,38].

Additionally, psychosocial factors are important in understanding the relationship between stress and CVD [39,40]. Experiencing major depressive disorder and depressive symptoms as a result of stressful life events is associated with an increased risk of heart failure, stroke, and peripheral artery disease, even controlling for traditional CVD risk factors [41]. This association is supported by both biological and behavioral mediators, including poor diet and sedentary lifestyle [42,43]. Post-traumatic stress disorder (PTSD) and other anxiety disorders have also been linked to CVD risk [39,44,45,46] through numerous biological and behavioral mechanisms, including dysregulation of negative affect [34].

Indigenous people suffer from PTSD, depression, anxiety, substance use and suicidal ideation at higher rates than other groups. As a group, Indigenous people have a long history of settler-colonist-induced trauma including forced relocation, economic, social, cultural and religious oppression, and genocide [47]. As a result, today, many Indigenous people continue to experience stress and trauma in their childhood and on into adulthood including discrimination and racism, both interpersonal and systemic, unemployment, poverty, incarceration, accidental injury, and interpersonal violence [48,49,50,51]. In this review, we will examine Indigenous-specific stressors and their effects on CVD and CMD. For Indigenous people, these include disproportionate experiences of historical trauma [52], early childhood trauma [53], and adult stress and trauma [54], compared to non-IP. Preliminary data suggest that stress has a strong relationship to CMD for Indigenous people; Indigenous patients with larger stress burdens are more likely to suffer from metabolic risk factors [55], T2D [56], and CVD [54,55,57]. We discuss how historical trauma, early childhood stress/trauma, and adult stressors contribute to cardiometabolic disparities for Indigenous people and how these health needs can be addressed to reduce cardiometabolic risk factors.

## 2. Methods

An Indigenous and non-Indigenous scholar team authored this manuscript. Indigenous authors include a PhD-level licensed behavioral health clinician and researcher, as well as two Indigenous physicians. Therefore, we aimed to use a decolonial lens to guide this project [58]. Specifically, we drew on personal experiences as Indigenous people, experiences working with Indigenous patients, and challenged Western scientific assumptions while consciously making room for Indigenous knowledge and perspectives. We also uphold the United Nations Declaration of Rights of Indigenous People. Specifically, Article 24 establishes that, “Indigenous peoples have the right to their traditional medicines and to maintain their health practices, including the conservation of their vital medicinal plants, animals and minerals. Indigenous individuals also have the right to access, without any discrimination, to all social and health services.” (UNDRIP, Article 24) [59].

**Figure 1:**
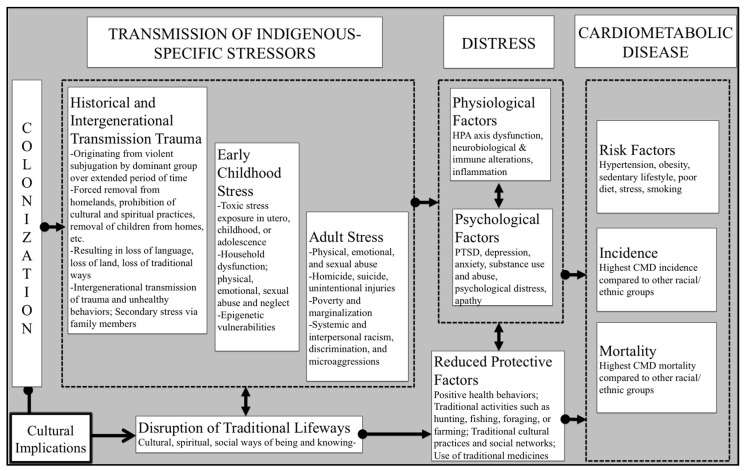
Indigenous stress–cardiometabolic disease pathways, adapted from Kinser and Lyon [60] and Warne and Lajimodiere [61]. HPA: Hypothalamus-Pituitary-Adrenal Axis, PTSD: Post Traumatic Stress Disorder, CMD: Cardiometabolic Disease.

We completed a non-systematic review of the literature with data collected until December 2020. The literature search had no location or age restrictions. Literature searches used the following words and their synonyms: Indigenous + [Early childhood stress or Historical Trauma or Stress] + Cardiometabolic Disease. An Excel spreadsheet was created to categorize articles. To accompany the review of literature, a figure (see Figure 1.) was created to demonstrate the relationships between Indigenous specific stressors and CMD noted in the literature. 

## 3. Historical Trauma and CMD

Historical trauma is the “cumulative emotional and psychological wounding over the lifespan and across generations, emanating from massive group trauma experiences” [62]. The trauma originates with subjugation of a population by a dominant group that deliberately and systematically inflicts physical and emotional violence on the targeted population over an extended period of time [63]. These traumatic events reverberate through the population and the effects of this stress persist across generations [52,64,65]. Thus, examining historical trauma provides an explanatory framework for understanding a wide range of health disparities with psychological, social, and biological mechanisms [63], ([66] p. 132), [67,68,69,70].

Traumatic experiences for Indigenous people include genocide, ethnocide, forced removal from their homelands, forced removal of children from their homes, and other forced assimilation policies obstructing or decimating traditional lifeways such as government and political systems, food systems, and family systems. Indigenous people also experience violations of treaty rights protecting land and human rights and are unlawfully incarcerated for defending their homelands and Indigenous ways of knowing and living. Today, Indigenous people think about such traumas often including loss of language, loss of land, and loss of traditional life ways: among Indigenous adults, almost half think about historical losses daily [71]. For Indigenous adolescents, approximately 20% report daily thoughts about historical loss [64]. These repeated and ongoing stresses comprise significant allostatic load, resulting in measurable health outcomes.

The health consequences of historical trauma have been examined among Indigenous people, as well as other groups that have experienced massive group trauma experiences, demonstrating a number of consequent physical and mental health concerns. For example, descendants of Holocaust survivors, African slaves in the US, Japanese Americans who had been interned during the second World War, and survivors of the World Trade Center attacks report high incidence of PTSD, dysregulation of HPA axis functioning, higher CVD mortality rates, and change in salivary cortisol levels [72,73,74,75].

Among Indigenous people, historical trauma is associated with increased anger [71], intimate partner violence [76], depression [64,71], suicidal ideation [62], negative self-image [62], anxiety [62], and substance use and abuse [52,65,71]. Further, participation in forced colonial assimilation programs, including boarding school, is related to worsened physical and mental health status such as anxiety, depression, PTSD, suicidal ideation and alcohol/drug abuse [77]. American Indian (AI) boarding schools attendees reported more health problems such as reduced physical functioning, physical limitations, pain, and overall poor health in comparison to AIs in the same age bracket that did not attend boarding schools [78,79].

Historical trauma is proposed to affect cardiovascular health for Indigenous people via a number of different mechanisms, including:

(1) Physiological Stress. One conceptualization of historical trauma is as an ongoing stressor that taxes the body physiologically, similar to other stressors. Specifically, this model proposes that historical trauma creates contemporary distress, which may be displayed through symptoms of PTSD, anxiety, or depression, taxes regulatory systems, including the HPA axis and balance between sympathetic and parasympathetic nervous systems, and results in the development of CVD risk factors, including insulin resistance [80]. In particular, Lefler and Belt illuminate that, “diabetes needs to be considered a manifestation of intergenerational PTSD” [80], p.73. This stress may additionally be transmitted intergenerationally through epigenetics, resulting in health risks being passed from parents to children through generations [59,81,82].
(2) Disruption of Food Systems, Medicines, and Traditional Lifeways. Through a variety of contributors (e.g., forced European farming methods, prohibition of traditional ecological methods and beliefs, forced relocation, introduction of European animals and plants, environmental degradation), Indigenous people have significantly different diet and exercise patterns compared to pre-colonization food activities including hunting, fishing, foraging, and traditional farming. Thus, prohibition of traditional foodways is directly linked to an increased sedentary lifestyle [83]. Government-sponsored food programs have replaced traditional foods that may have protected Indigenous people from metabolic disease [83], including tepary beans (Tohono O’odham), salmon (Yup’ik) [84] and Jerusalem artichoke and persimmons (Cherokee) [85], with foods that are high in simple sugars and saturated fats [86], leading to increased rates of obesity, hypertension, diabetes, and heart disease [87]. Additionally, remedies and ceremonies used in traditional medicine to treat diabetes and heart disease, such as the use of hawthorn or sumac leaves to lower blood pressure and glucose [88,89,90], were prohibited in the US until recently (Indian Freedom of Religion Act of 1978), resulting in the carry-over of stigma against practicing traditional spirituality today [91]. One study specifically implicated the loss of traditional Indigenous CMD prevention practices among Pacific Northwest AIs in the effect of historical trauma on increased risk for CMD [92].
(3) Intergenerational Transmission of Stress and Trauma. Through a process called trauma transmission, family members or those with close relationships to someone with PTSD may develop symptoms that mirror PTSD without any primary traumatic experience, called secondary stress [93]. Specifically, in marital and parent–child relationships, as partners or children learn about the traumatic event, they emotionally experience the event and begin to mirror the symptomology of the PTSD victim [94]. The trauma transmission model [95] specifies that partners use not only sympathy, but empathy for their partner’s experience, resulting in a realistic experience of the trauma themselves. Therefore, the closer the relationship, the more at risk one is for developing secondary trauma, especially for those living together. While most research on secondary trauma is in military families, researchers have begun to look to this model as an explanatory model for the passing of trauma and trauma symptoms to subsequent generations of Indigenous families [96].
  (3a) Intergenerational Transmission of Poverty. Colonization aimed to prevent and terminate Indigenous lifeways including community, family, and parenting values and practices to make way for Western European ideals. For instance, residential schools aimed to shift Indigenous parenting styles due to military and religious influences. In addition, residential schools purposefully created a low socioeconomic class of Indigenous people given that these schools trained students to work in the lowest-paying jobs available. Today, the experience of poverty, including homelessness and lack of unemployment, are both prevalent for Indigenous populations and relate to negative health outcomes [97,98]. It is important to note that poverty is also intergenerational. The intergenerational transmission of poverty describes the pattern of parental poverty experienced by a child persisting into the child’s own adulthood, which is influenced by a complex set of positive and negative factors such as private transmission of capital (e.g., inheritance of land and money), public transmission of resources (e.g., low investment in education of low-income neighborhoods), and behavioral traits (e.g., learned habits around wealth accumulation) from one generation to the next. Parental poverty status influences children’s resiliency and human capital development (e.g., educational performance and social development), contributing to the intergenerational transmission of poverty. Additionally, the costs of ill health and healthcare may be enough to ensure persistent poverty and are a driver of downward economic mobility demonstrating a bidirectional association between poverty and health status [99]. Therefore, it appears that health status and poverty may be linked through intergenerational transmission of trauma experiences for Indigenous people.
  (3b) Intergenerational Transmission of CMD Risk Behaviors. The stress of experiencing increased thoughts of historical trauma and loss are additionally related to an increase in CMD risk behaviors. For example, mothers are more likely to smoke during pregnancy if their mother or grandmother attended residential school compared to those who did not have family member residential school attendance [100], which puts babies at risk for low birth weight and subsequent cardiometabolic disease [101,102]. In addition, violence and militaristic parenting styles were taught through colonial practices including boarding schools. Therefore, intergenerational trauma may be perpetuated by learned harmful parenting techniques and worsened parent–child relationships, which negatively affects the child via the adverse childhood experiences mechanism [96]. This may lead the child to adopt personal unhealthful behaviors, including dysfunctional parenting practices, which in turn leads to adverse childhood experiences in their offspring.

Historical trauma is associated with increased risks for mental and physical health risks among Indigenous people. There is evidence that pathways through physiological and mental health mechanisms as well as intergenerational trauma and trauma transmission models explain the relationship between historical trauma and health outcomes. However, all require more investigation. While little research is currently available investigating CMD as a specific outcome of increased historical loss, there is support for a potential relationship given the links between historical trauma and worsened physical and mental health status.

While this section is focused on the health effects of historical trauma amongst Indigenous populations, it is important to emphasize that Indigenous people are resilient and are thriving in the face of the trauma they have, and continue to, experience. While we will speak more to the research on the implementation of Indigenous culture and its positive health effects in the upcoming section titled, *Existing Interventions and Actionable Solutions*, it is important to clarify that many health disparities that Indigenous people experience are now considered a result of colonization and its effects, while health-related resilience is related to positive cultural and tribal relationships and activities.

## 4. Early Childhood Stress and CMD

Indigenous children and youth suffer from more stress and trauma compared to their non-Indigenous counterparts, which may set the trajectory for adulthood illnesses such as PTSD and cardiometabolic disease [53]. These early traumas not only predict mental health outcomes in adulthood; they predict physical illnesses as well. Stress experiences can physiologically change developing organs, including the heart, before birth [101,103], during childhood [104], and into adulthood [105], leaving stress victims more susceptible to CVD. This is particularly true of individuals who have experienced adverse childhood experiences (ACE), including various forms of maltreatment (bullying, exposure to crime, discrimination, etc.), physical, emotional, and sexual abuse, family substance abuse, parental divorce, neglect, and other forms of household dysfunction due to adult mortality and morbidity [24,106]. In a landmark study of more than 17,000 adult patients from a Health Maintenance Organization in California, researchers showed a strong association in the dose-response relationship between ACE scores (the number of categories experienced by an individual before age 18 years, not the number of unique experiences) and health-averse behaviors and disease. Furthermore, individuals with higher ACE scores tend to have lower graduation rates and academic achievement, which correlates with both reduced socioeconomic status and greater health risks. The proposed etiology includes altered neurodevelopment with subsequent social, emotional and cognitive impairment, which in turn leads to the adoption of risky health behaviors, such as smoking, substance use, alcohol abuse, leading a sedentary lifestyle, and overeating. The relationship between ACE score and CVD and its risk factors is particularly strong [25,107,108,109]. Compared to individuals without ACEs, those with an ACE score of 3 or more are over two times more likely to smoke, one and a half times more likely to be severely obese or have T2D, and twice as likely to have CVD [23,110].

AIs experience a disproportionate ACE burden. A recent study of 516 AI adults from South Dakota documented an ACE score of 3 or more in 45.4% of participants compared to 17.4% of region-matched non-AI controls, and high ACE scores were associated with increased current, self-reported smoking status [111]. Other studies show that AIs are 5 times more likely than non-AIs to experience 4 or more ACEs, increasing the risk of CVD three-fold [112]. According to National Survey of Children’s Health data, AI children and adolescents less than 18 years of age were more likely to have higher ACE scores when compared to non-Hispanic White children; 26.8% of AI youth had 3 or more ACEs compared to 11.5% of non-Hispanic White children [113]. These studies and others detail a high prevalence of emotional and physical neglect, substance abuse and incarceration among AI family members, as well as children witnessing domestic violence [114]. An investigation of First Nations adults found that experiences of discrimination and lack of social support in early life contribute to symptoms of depression, suggesting that stressors may have a compounding effect [115].

In addition to drastically high rates of ACEs, Indigenous children commonly experience discrimination in social and government settings and programs. For example, in schools, Indigenous students do not receive culturally appropriate education [116,117], which is related to academic outcomes [118]. In addition, Indigenous students are more likely to have novice or unqualified teachers, not have access to math and science classes, and be expelled or suspended [119] compared to non-Hispanic White students. Indigenous youth are over-represented in the juvenile justice system and more likely to receive harsher sentences and more punitive punishments, such as restraints or isolation, compared to other racial and ethnic groups [120].

Similar to educational settings, social services have historically (e.g., boarding schools) and currently demonstrated pervasive discrimination towards Indigenous children and families. AI children are removed from their homes 13 times more than White children and placed in foster care twice as often [121,122], which exacerbates cardiometabolic risk and increases the prevalence of obesity [123,124]. Strength and stability of family relationships are critical aspects of health and well-being for children. For example, toddlers who showed insecure attachment to their mothers at age 2 had a 30% increased risk of obesity by age 4 1/2 [104]. Young children with objectively-measured poor-quality mother–child relationships had 2.45 times increase in prevalence of adolescent obesity compared with those with good-quality mother–child relationships [104].

In addition to childhood stress and trauma, stress during the perinatal period—from gestation to early infancy—contributes to CVD risk in children. During intrauterine life, ongoing processes of epigenomic modification, intimately associated with growth and development, result in multiple vulnerable periods for environmental factors to influence these processes [125,126]. This phenomenon is well illustrated with maternal obesity correlating with offspring obesity and cardiometabolic risk [127,128,129,130,131]. Other than increasing the chance of persisting childhood obesity, this likely has long-lasting effects on offspring metabolism and glucose homeostasis as well as pancreatic function, leading to T2D. In addition, there seem to be elaborations of cortisol with subsequent inflammation and alterations in vascular function with the subsequent development of hypertension [106,132]. While research examining perinatal insults, and later CVD, among AIs is limited in general, one such relationship was shown in American Indian/Alaskan Natives (AI/ANs) [133]. In an innovative study from Wisconsin, excessive maternal weight gain in pregnancy as well as neonatal macrosomia (indicating possible insulin resistance during gestation) was associated with obesity at 1 year of age in AI infants [134]. Furthermore, the BMI category at age 1 year tracked into the children’s school-age years, providing direct evidence of untoward fetal determinants of CVD risk in young AI children. Intrauterine exposure to T2D also seems to increase obesity as well as T2D risk in AIs [135]. These data suggest a substantial component of cardiometabolic disease risk has a prenatal basis [136,137,138,139,140,141].

There is also unique CVD risk associated with stress conferred in infants. Preterm birth disproportionately affects AI/AN women [142], which increases the risk for infants to develop obesity as they age [143], which may be related to feeding protocols employed to achieve neonatal weight gain [144]. Alarmingly, maternal stressful life events increase the risk of preterm birth by approximately 2.5 times [101,103]. Since AI women experience more emotional abuse (66.4% versus 52.0%), domestic violence (55.5% versus 34.5%), and sexual violence (56.1% versus 49.7%) compared to non-Hispanic White women [145], their children are more vulnerable to preterm birth.

There are a number of ways in which Indigenous infants, children and youth are placed at higher risk for developing CMD later in life. More scholarly work is needed to trial and evaluate interventions that obviate this risk by breaking the negative cycle of stress in utero and in childhood that increases the risk of CMD morbidity and mortality. The unexplored developmental, social and relational factors related to adult CMD might be an essential key to understanding and preventing CMD amongst Indigenous populations [146].

## 5. Adulthood Stress and Trauma and CMD

AI/ANs experience psychological and emotional trauma at much higher rates than other racial/ethnic groups in the US: estimates of the prevalence of lifetime trauma among AI/ANs range from 60% to 80% [147], which compares to an estimated lifetime exposure to trauma among the US general population that ranges from 50% to 60% [148]. These traumatic events include being a victim of a disaster or life-threatening accident; serving in direct combat; being physically, sexually, or emotionally abused; or witnessing others being raped, injured, or killed [54,145,149]. Native women suffer sexual violence at the highest rate of any racial or ethnic group in the US and are almost three times more likely to be killed by an intimate partner compared to non-AI/AN women [145,150,151,152,153]. The vast majority of the sexual violence experienced by AI/AN women is committed by non-Native perpetrators [152], consistent with the devastating history of rape and sexual assault associated with colonization [154,155]. Indigenous men are more likely than any other group to be killed by police [156]. Finally, Indigenous men and women are disproportionately represented within the criminal justice system as an ongoing legacy of colonization [157,158,159].

These experiences of trauma are important in the subsequent development of PTSD among AI/ANs [111], with combat experience and interpersonal violence cited most often as leading causes of PTSD and related symptoms [160]. AI/ANs are additionally twice as likely as the general population to develop PTSD, which has been proposed to be the result of higher likelihood of experiencing trauma, rather than a higher conditional rate of developing PTSD [147,153,161,162].

Consequently, both the experience of trauma and the development of PTSD have been linked to CVD and hypertension through a myriad of biological, behavioral, and psychosocial pathways, although the majority of this research has been done in non-Indigenous populations [57,163,164,165,166]. However, a number of studies have focused on the relationship between trauma, PTSD, and CVD among AI/ANs, specifically [167]. For example, in the American Indian Services Utilization, Psychiatric Epidemiology, Risk and Protective Factors Project (AI-SUPERPFP), over two-thirds of participants on a Southwest and a Northern Plains reservation had experienced significant traumatic events, and PTSD was positively correlated with obesity, self-reported hypertension, and CVD [54,55]. Additionally, PTSD fully mediated the relationship between trauma experienced before age 13 and biomarkers of allostatic load, including cardiovascular, neuroendocrine, inflammation, and metabolism measures [168]. In another large sample, traumatic stress was associated with cardiovascular disease [169], as well as higher HbA1c levels among those with diagnosed with T2D, suggesting poorer T2D control [170]. Thus, rising CVD rates among AI/ANs may be better understood if traumatic experiences, along with PTSD diagnosis, are considered along with other traditional CVD risk factors.

In addition to extreme traumatic events prompting the development of PTSD, Indigenous people experience ongoing discrimination in many social contexts. Discrimination refers to the differential or unfair treatment experienced by individuals or groups because of a devalued stigmatized or group identity [171] that are rooted in colonization [172]. Discrimination experienced at the interpersonal level is perpetuated by negative attitudes and stereotypes of IP as drunk, dirty, primitive, and homogenous begin as early as childhood education [173,174,175,176,177,178,179]. These negative perceptions also include that Indigenous people no longer exist, are “people of the past,” and get things for free [180], are perpetuated in media. Native voices are silenced through lack of representation and paraphrasing Native experiences by White narrators [181], misappropriating cultural values by commercially producing Native regalia, plant medicines, and artwork, and the continued use of racist mascots, for example [182]. These representations provide a limited and incorrect view of the many cultures, values, and practices of more than 570 federally-recognized tribes and negatively affect the self-efficacy, feelings of community worth, and achievement-related possible selves of AI/ANs who witness them, often on a continual basis [182].

These experiences of discrimination effect cardiometabolic health in a number of ways. A large body of research implicates the repeated daily experiences of discrimination as both acute and chronic stressors, which increase allostatic load and have long-term health consequences [183]. Among IP around the world, discrimination and microaggressions have been associated with a higher likelihood of self-reported diabetes among southwest AIs [56], increased diabetes distress and higher blood pressure in AI from the northern Midwest [184,185,186], higher self-reported CVD (including heart attack and stroke) among Maori in New Zealand [187] and CVD diagnosis among the Indigenous Sami population in Norway [188].

Additionally, negative emotional states, including depression, anxiety, anger, and hostility, have also been proposed to act as a mechanism for discrimination’s negative effect on health [189]. The experience of discrimination among Indigenous people is associated with depression [186,190,191], anxiety [192], anger and aggression [193,194] and more general mental health concerns [187,195]. Mental health appears to function as a mediator between negative life experience and CMD risk, possibly due to severity of life experience or lowered coping mechanisms that lead to reduced positive health behaviors or significant damage to physiological functioning. Although we focus this discussion on the effect of experiences of discrimination primarily among adults, Indigenous people experience discrimination not just in adulthood but beginning in childhood, which has an effect on the accumulation of allostatic load through the lifespan [115,196,197]. Thus, it is important to consider the cumulative effect of daily experiences of discrimination across all stages of life.

In addition to daily experiences with discrimination, a legacy of racial discrimination persists within the medical domain, including the forced sterilization of more than 3000 AI/AN women ages 15–44 years between 1973 and 1976 [198,199] and unethical research practices. A contemporary example involves unapproved genetics research conducted without knowledge or the consent of tribal member participants on blood samples originally collected from the Havasupai Tribe for the purpose of studying high rates of diabetes [200]. This legacy of discrimination has informed modern day healthcare-seeking practices and has resulted in increased distrust of the medical system and medical professionals among Indigenous people and decreased quality of care due to persistent biased beliefs of providers in the medical system [172,201,202].

Biased beliefs of medical providers include negative stereotypes of AI/ANs as alcoholics or drug seeking, more challenging and less compliant than White patients, unmotivated, and ignorant [203,204,205], and in one study, 84% of medical providers reported preferring to treat White patients rather than AI/AN patients [203]. Many medical providers report being unaware of the cultural practices and context of AI/AN patients [205]. These attitudes damage the provider–patient relationship and increase patient stress [51]. Thus, AI/ANs report more barriers to healthcare access, including issues related to language, culture, discrimination and trust, than White patients [206,207]. Specific examples include patients experiencing threats, being dismissed, their medical problems and pain minimized and these experiences result in receiving worse care [208]. Systemic bias results in the underutilization of preventive medical care, including breast and cervical cancer screenings [209], cholesterol and hemoglobin A1c testing [210] and medical adherence in diabetes management [211], which results in poorer overall health [212].

In conclusion, discrimination and trauma in daily life and in interactions with the medical system contributes to the chronic accumulation of stress and the development CVD through several mechanisms. Addressing the cumulative effect of acute and chronic stressful experiences throughout adulthood, including both discrimination and trauma, is essential in understanding disparities in CVD and related risk factors that impact the health of Indigenous people.

## 6. Existing Interventions and Actionable Solutions

Given the existing CMD disparities experienced by Indigenous people, a number of interventions have focused on improving the health of Indigenous people with an emphasis on CMD. For example, the Together on Diabetes project was targeted to rural AI/AN youth with or at risk of T2D [213,214], incorporating a home-based curriculum including information about nutrition, physical activity, and healthy living goals, support from program personnel to sustain healthy behaviors over 6 months, and group community activities, including fun walks and food demonstrations. Other randomized controlled trials targeting Indigenous adults with T2D or other CVD risk factors have been reported or are ongoing [215,216,217,218]. However, these interventions do not employ a community based participatory research model to sufficiently incorporate cultural elements that address Indigenous-specific stressors and needs of the participants [219,220]. The following section reviews interventions that have been developed to address CMD by neutralizing the effects of historical trauma through decolonization and revitalization, supporting young Indigenous families, and providing equitable life experiences for Indigenous people in our society. Additionally, supplementing the toolkit provided by the Special Diabetes Program for Indians [221] housed in the Indian Health Service, we provide suggestions for developing new programs or interventions that aim to reduce CMD in Indigenous communities.

### 6.1. Addressing Historical Trauma

Interventions that focus on reducing the pathogenic effects of historical trauma use a holistic, culturally relevant approach to encourage community empowerment and address symptoms of historical trauma from a Native perspective. A sense of cultural connection is especially critical for Indigenous peoples, as it relates to positive health and well-being, brings meaning to one’s life and increases motivation for health and well-being [222]. For Indigenous people, traditional and cultural activities do not take place in isolation, but in relation to others [223]. The act of colonization sought to break down relationships of Indigenous peoples by prohibiting communal and intergenerational living, forcibly removing young children from their homes and placing them into boarding schools, and allotting land only to nuclear families. The act of decolonizing [60] includes revitalizing and reconnecting to family, community, and tribe [224].

Cultural revitalization improves the health and well-being of Indigenous people. One literature review of the role of traditional cultural participation in health and well-being outcomes around the world found that those tribal members that used their traditional language more often had improved high school graduation rates (Hawaii), decreased suicide rates (Canada), reduced smoking (US Plains and Southwest tribes), reduced health risks (Australia), improved health (Hopi), and lower rates of diabetes (Canada) [225]. Other studies have noted that connection to and immersion into traditional culture, such as attending cultural ceremonies and use of traditional language, is associated with a decrease in metabolic disease-related risk factors including depression and suicidality [226,227], diabetes, smoking, and obesity [228,229,230].

Accordingly, successful programs first center their interventions through an Indigenous lens of the disease (e.g., diabetes) and an Indigenous view of the solution. In one example in the Coast Salish population, traditional plants for food and medicine were administered to address diabetes in the community [231]. Foraging for the plants increased physical activity and built bonds between family and community members. A randomized controlled study with a Southwest tribe in the US compared a less structured tribal-specific cultural intervention involving learning tribal history, language, and crafts to a standard physical activity and diet intervention. Tribal participants in the cultural arm achieved better weight loss success and lower blood glucose levels in a 2 h oral glucose challenge than their counterparts who had participated in the standard condition [232]. In another randomized controlled study, traditional cultural practitioners taught Hula dance and cultural teachings to one group for 12 weeks [233]. The intervention group had a significant reduction in systolic blood pressure compared to a waitlist control group from baseline to 3 months post-intervention. In other studies, culturally adapted programs developed in conjunction with tribal representatives have resulted in significant reductions in weight, cigarette smoking, blood pressure, and blood lipids among Indigenous people [160,234,235]. Thus, interventions that center traditional Indigenous culture, activities, nutrition, and language and are developed in collaboration with tribal members are effective in addressing CMD and CVD risk factors present in Indigenous communities [236,237].

To develop new interventions, it is essential to collaborate and put culture first. Interventions should be developed in conjunction with tribal communities and incorporate elements of local tribal culture, including language and ceremony, native foods and nutrition, traditional activities, such as, beadwork, weaving, hunting, gardening, and gathering berries, herbs and other traditional plants used for food or ceremonies, and learning tribal history. Cultural interventions envisioned and implemented with an Indigenous lens redress the effects of colonization by revitalizing traditional lifeways to address the harmful effects of historical trauma and improve CMD risk and outcomes. As a consequence, restoring tribal practices and community values may improve self-efficacy, cultivate a sense of belonging, strengthen identity, and provide valuable social and other emotional support.

### 6.2. Reducing Childhood Stressors

The most effective interventions to attenuate the effects of childhood stress and trauma focus on improving prenatal care and parenting support and skills. Home-visiting programs involving prenatal and infancy home visits to at-risk women delivered by a medical health paraprofessional are well-established, evidence-based practices that improve parenting, maternal life outcomes, and children’s health and behavior outcomes into adulthood [238]. For Indigenous people, offering culturally safe home-visiting programs may improve outcomes compared to non-tailored programs. Wind River’s Family Spirit home-visiting program incorporates history and cultural traditions so participants “can better manage the effects of historical and intergenerational trauma on their daily behaviors” [239].

An early randomized controlled trial tested a home-visiting program supplemented by culturally adapted lessons taught by bilingual AI women on prenatal care, labor, delivery, breastfeeding, home safety, immunizations, family planning, sexually transmitted disease prevention, and maternal goal setting for personal and family development. Navajo and Apache pregnant teens in the intervention arm showed improvement in knowledge, maternal skills, and involvement, relevant to the active control group [240]. This program was developed into the Family Spirit Home-Visiting Program and is now associated with multiple tribal health programs throughout Indian country. Implementations of the program have been shown to improve parenting outcomes, including fewer depressive symptoms, externalizing problems, and substance use, as well as child outcomes, including externalizing and internalizing behaviors [146,241,242,243]. Other culturally-adapted home-visiting programs have been successful in improving outcomes related to CMD in Indigenous communities in the US, Canada, South Africa, and New Zealand [244,245]. As a result of the Maternal Infant and Early Childhood Home-Visiting Program (MIECHV) authorized by the US Congress in 2010 and reauthorized in 2018, which specifically allocates money for home-visiting programs in tribal communities, Tribal MIECHV programs have been funded in 14 states [246].

In addition to improving the stability of the family through social and emotional safety, economic stability may also play on important role in child well-being. A novel study amongst the Eastern Band of Cherokee Indians found that economic stability was related to improved child mental health. In particular, income supplements that moved American Indians out of poverty were associated with reduced psychiatric symptoms of children [247]. Therefore, it is possible that the combined experience of culturally relevant social, emotional and economic safety for Indigenous children place them on a trajectory for improved health. Health status is correlated with social and economic status for American Indian people and should be considered for population-level interventions [248].

To address the development of CMD and related risk factors, it is essential to consider psychosocial health throughout the lifespan, especially prenatal health and child development. Interventions considering the effects of childhood stress and trauma should focus on prenatal care and improving parenting skills through a variety of means, which may include nurse home visits, parenting classes, social parenting groups or involvement in cultural activities. Psychological and emotional health should be addressed using tribally specific beliefs, and both the development and evaluation of such programs should include knowledge keepers, including elders and/or traditional healers in a community-based participatory approach.

### 6.3. Reducing Trauma and Discrimination

Colonization and marginalization continue to perpetuate discrimination and inequities, causing stressful and traumatic experiences of Indigenous people. Discrimination towards Indigenous people exists in many systems including education, justice, social services, housing and employment, negatively affecting the life prospects of Native people as well as their health and well-being. Therefore, effective interventions to address discrimination will likely require a multi-faceted, multi-layered approach involving individual-, societal-, and political-level changes.

Solutions include (1) increasing equitable treatment in social spaces, i.e., the ability to practice cultural lifeways without punishment, and (2) eliminating biased beliefs that lead to discrimination. This involves education, training, and regulations and consequences for discriminatory behavior throughout social systems. Within education, several different government entities and universities have begun to enact policies to educate their students about Indigenous people to reduce bias and stereotypes. In 1998, Minnesota became the first US state to implement a requirement that AI/AN history be incorporated into the K-12 education curriculum [249]. As of 2015, there were a total of nine states with such legislation. However, low descriptive representation and minimal campaign contributions may be limiting other states’ interest in similarly inclusive educational policies [249]. Several universities in Canada, such as the University of Winnipeg, have followed suit requiring an Indigenous studies course to graduate. New Zealand includes Māori-medium education in primary and secondary schools, which includes Indigenous curriculum specific to the Māori taught mostly in the Māori language [250]. A systematic education is the foundation needed to reduce bias and discrimination against Native people [251].

Within healthcare settings, Indigenous people experience bias and discrimination that results in worsened care and health outcomes [208]. Cultural safety includes, “the delivery of quality care through changes in thinking about power relationships and patients’ rights” [252]. To this end, several programs exist that educate medical students about Indigenous health using collaborative and decolonizing methods [253,254,255,256], resulting in improved Indigenous health knowledge [257], improved knowledge about Indigenous perspectives, commitment to rural and underserved areas [255], and improved cultural responsiveness within healthcare settings [254].

Other programs exist within medical education that require competencies in social justice beyond just course completion [258,259,260]. Student competencies create a standard which all students must meet increasing accountability for students and require faculty to become more familiar with social justice content [261,262]. Indigenous core competencies, along with pedagogical principles have been created and implemented in Australia and New Zealand [263]. These principles go beyond the classroom and move into other spaces such as interpersonal behavior, mission statements, administration, hiring practices, hidden curriculum, and the environment and values of an organization.

Thus, to address increased CMD risk experienced by Indigenous people, it is essential to educate the public as well as healthcare providers, staff, and administrators about Indigenous peoples. Training must incorporate Indigenous culture and history to reduce bias, stereotyping, and discrimination of Native people in the medical setting and to improve providers’ ability to effectively treat systemic trauma experienced by Indigenous people [264,265]. Both culturally aware health education and increased accountability for engaging in all forms of bias and discrimination towards Indigenous people must increase at the individual, community, and national levels. Further, any such curriculum must include incorporating traditional Indigenous thoughts and actions into colonized spaces to increase equity and decrease discrimination (for examples, see Martinez [251], Dumbrill and Green [264], Burnette and Figley [265], Belone, Orosco [266]). It is also essential to address a significant barrier to successful systems changes, namely, the lack of appropriate funding, including underfunding of the pre-paid healthcare systems for AI/ANs, the Indian Health Service (IHS). Despite several changes to legislation in the 20th century, the IHS continues to go underfunded, which results in reduced and worsened health services [267].

## 7. Conclusions

Stress is an important factor for understanding and eliminating CMD disparities for Indigenous people. The burden of stress and trauma that Indigenous people experience is substantial; Common forms include settler-colonial historical relations, adverse fetal and childhood experiences, and adulthood experiences of stress, trauma, and discrimination is substantial. These experiences appear to put Indigenous people at a greater risk for CMD. While further investigation into these pathways is needed, promising interventions exist to reduce CMD risk in Indigenous communities. Successful interventions to reduce CMD incorporate culture, history, and needs of Indigenous people and are developed in tandem with the communities they are intended to serve or solely within the tribal communities themselves.

## Data Availability

Data sharing not applicable.
